# Field Diagnosis of Potato Nitrogen Nutrition Using a Bayesian Critical Nitrogen Dilution Curve and Canopy Spectral Sensing

**DOI:** 10.3390/plants15121868

**Published:** 2026-06-16

**Authors:** Jing Yu, Yonglin Qin, Li Li, Yang Chen, Liguo Jia, Mingshou Fan

**Affiliations:** 1 College of Agronomy, Inner Mongolia Agricultural University, Huhhot 010019, China; yujjing@imau.edu.cn (J.Y.); imauqyl@imau.edu.cn (Y.Q.); jialiguo@imau.edu.cn (L.J.); 2 College of Agronomy and Life Sciences, Shanxi Datong University, Datong 037009, China; 03120011@sxdtdx.edu.cn; 3 College of Resource and Environment, Inner Mongolia Agricultural University, Huhhot 010011, China; chenyang@imau.edu.cn

**Keywords:** potato, nitrogen nutrition index, critical nitrogen dilution curve, Bayesian hierarchical model, canopy spectral sensing, ratio vegetation index, precision nitrogen management

## Abstract

Accurate diagnosis of potato nitrogen status is critical for optimized fertilizer management and sustaining productivity. We used data from nine field experiments (2010–2018) across major potato-producing regions in northern China to develop a regional critical nitrogen dilution curve via a Bayesian hierarchical model. The curve, Nc = 4.179 × DW^−0.417^ (DW = whole-plant dry matter), provided the basis for calculating the nitrogen nutrition index (NNI), which was related to canopy spectral indices from a GreenSeeker sensor. Relationships between spectral indices and NNI were strongly growth-stage dependent. The tuber initiation–bulking period, approximately 29–70 days after emergence (DAE), represented the effective phenological window, with 29–55 DAE as the primary operational window for quantitative spectral diagnosis. Stage-specific ratio vegetation index (RVI) showed the most consistent association with NNI, whereas pooled whole-season models had low predictive power. The Bayesian framework quantified uncertainty, emphasizing that near-threshold NNI values require cautious interpretation. The resulting regional-average reference supports rapid field diagnosis of potato N status while accounting for cultivar, year, and site variability. These findings provide practical guidance for stage-specific N management and demonstrate the importance of growth-stage-aware spectral assessment in operational decision-making.

## 1. Introduction

Potato (*Solanum tuberosum* L.) is an important food, vegetable, and industrial crop worldwide. Nitrogen (N) supply plays a central role in canopy development, dry matter accumulation, tuber formation, and quality formation in potato production. Insufficient N supply restricts leaf area expansion and photosynthetic production, whereas excessive N application may delay crop maturity, reduce N use efficiency, and increase the risk of N loss to the environment. Therefore, developing reliable diagnostic methods for crop N status under diverse ecological and management conditions is essential for improving N fertilizer management in potato production [1,2,3,4].

The nitrogen nutrition index (NNI) is a widely used indicator for assessing crop N status based on the theory of critical N dilution. This theory assumes that plant N concentration declines with increasing biomass because of changes in structural tissue proportion and the development of storage organs. At a given level of crop biomass, the minimum plant N concentration required to sustain maximum growth is defined as the critical N concentration (Nc) [5,6]. NNI is commonly calculated as the ratio of actual plant N concentration to Nc, and it can be used to distinguish N deficiency, optimal N supply, and excessive N status. Bélanger et al. developed a critical N dilution curve for potato in eastern Canada and demonstrated the usefulness of NNI for diagnosing potato N nutrition [1]. Subsequent studies have shown that the parameters of potato critical N dilution curves may vary with cultivar, environment, sampled organ, and crop management [2,3,7]. Therefore, directly applying a curve developed for a specific region or cultivar may not be appropriate for regional-scale N diagnosis.

In recent years, research on critical N dilution curves has moved from single-site empirical fitting toward multi-environment data integration and uncertainty analysis. Makowski et al. proposed a Bayesian framework for estimating the parameters of critical N dilution curves while explicitly quantifying their uncertainty [8]. Ciampitti et al. compiled a global dataset for parameterizing critical N dilution curves across major crop species, highlighting the value of multi-environment datasets for improving model generality [9]. Lacasa et al. compared statistical approaches for fitting critical N dilution curves and showed that hierarchical Bayesian methods are particularly useful when data are obtained from complex or unbalanced experimental structures [10]. For potato, Bohman et al. used a partially pooled Bayesian hierarchical approach to quantify variation in critical N dilution curves under genotype × environment × management interactions [11]. These studies indicate that Bayesian hierarchical modeling can provide a more robust framework for developing regional diagnostic references when data are collected across years, sites, and cultivars.

Although NNI has strong physiological relevance, its conventional determination requires destructive plant sampling, drying, weighing, and laboratory N analysis. These procedures are time-consuming and limit the use of NNI for in-season field decision-making. Canopy spectral sensing provides a practical alternative for rapid, non-destructive assessment of crop N status. Vegetation indices based on red and near-infrared reflectance, such as the normalized difference vegetation index (NDVI) and ratio vegetation index (RVI), are closely associated with canopy chlorophyll content, leaf area, and biomass, and have been widely used for monitoring crop growth and N status [12,13,14]. In potato, proximal active sensors, unmanned aerial vehicle imagery, and satellite remote sensing have all been explored for monitoring N status and predicting crop performance. However, previous reviews have emphasized that model transferability, growth-stage dependence, and the need for ground validation remain major constraints for practical application in precision N management [15,16,17,18].

Two approaches are commonly used to estimate potato NNI from spectral information. The first is an indirect approach, in which plant N concentration and biomass are estimated separately from spectral indices and then combined with a critical N dilution curve to calculate NNI. The second is a direct approach, in which empirical relationships are established between spectral indices and NNI. The indirect approach has a clearer physiological structure, but errors may accumulate across multiple modeling steps. The direct approach is simpler and more suitable for rapid field diagnosis, but its accuracy depends strongly on crop growth stage, canopy structure, and index selection. Recent work integrating UAV multispectral imagery, machine learning, and Bayesian critical N dilution curves has shown the potential of combining physiological N diagnosis with remote sensing to characterize spatial and temporal variation in potato NNI [19]. Nevertheless, in practical field management, proximal active canopy sensors remain attractive because of their lower operational complexity, lower cost, and reduced dependence on illumination conditions.

The major potato-producing areas in northern China are characterized by substantial variation in soil type, climate, cultivar, and N management. Previous studies have examined critical N dilution curves for potato under different seasonal conditions in China; for example, Bai et al. compared whole-plant and organ-specific curves for spring and autumn potato [20]. However, regional NNI diagnosis based on multi-year, multi-site data and linked with proximal canopy spectral sensing remains limited for northern China. Therefore, the objectives of this study were to: (i) develop a regional critical N dilution curve for potato using a Bayesian hierarchical model based on nine field experiments conducted from 2010 to 2018 in northern China; (ii) evaluate the direct estimation of NNI using NDVI, RVI, and their time-normalized forms; and (iii) identify the growth-stage window in which canopy spectral indices can reliably diagnose potato N nutrition. This study aims to support rapid field diagnosis of potato N status and provide a basis for sensor-assisted precision N management.

## 2. Materials and Methods

### 2.1. Experimental Sites and Design

Nine field experiments were conducted from 2010 to 2018 in major potato-producing areas of Inner Mongolia, northern China, including Wuchuan County (41°05′39″ N, 111°27′01″ E; 1618 m a.s.l.), Linxi County (43°35′57″ N, 118°03′12″ E; 800 m a.s.l.), Chahar Right Middle Banner (41°16′08″ N, 112°37′50″ E; 1715 m a.s.l.), and Hanggin Rear Banner (40°53′10″ N, 107°08′01″ E; 1052 m a.s.l.). The experimental sites covered typical temperate continental monsoon climatic conditions and major soil types in the region, including chestnut soil and aeolian sandy soil. The basic soil properties of the 0–20 cm layer before planting are shown in Table 1.

Each experiment was arranged in a randomized complete block design with four replicates. To generate a wide range of crop N status, N fertilizer rates ranged from 0 to 450 kg N ha^1^ across experiments (Table 2). Two potato cultivars, ‘Shepody’ and ‘Kexin 1’, were used. These cultivars were commonly grown in the study region during the experimental period and represent important cultivar types in local potato production. Phosphorus and potassium fertilizers were applied before planting at 180 kg P_2_O_5_ ha^−1^ and 300 kg K_2_O ha^−1^, respectively, using calcium superphosphate and potassium sulfate. Ridge cultivation and drip irrigation were adopted, and all other field management practices followed local high-yield production practices to ensure that N supply was the main limiting factor for crop growth.

### 2.2. Data Collection

#### 2.2.1. Whole-Plant Dry Matter and Plant Nitrogen Concentration

Plant samples were collected at key growth stages, including the seedling stage, tuber initiation stage, tuber bulking stage, and starch accumulation stage, according to the sampling dates listed in Table 2. Representative plants were sampled from each plot and separated into roots, stems, leaves, and tubers. Fresh samples were heated at 105 °C for 30 min and then oven-dried at 80 °C to constant weight. The dry weight of all plant parts was summed to calculate whole-plant dry matter (DW, t ha^−1^). Dried plant samples were ground and passed through a 1 mm sieve for chemical analysis. Plant nitrogen concentration (PNC, %) was determined using the Kjeldahl method [21].

#### 2.2.2. Canopy Spectral Reflectance Measurements

Canopy spectral reflectance was measured at the same time as plant sampling using a GreenSeeker active canopy sensor developed by Oklahoma State University, USA. Measurements were conducted under wind-free conditions. In each plot, areas with uniform plant growth and no missing plants were selected. The sensor was held 60–80 cm above the potato canopy and moved steadily along the rows. Data were recorded using the instrument-connected PC. Each plot was measured nine times, and the average value was used for subsequent analysis. The measurement dates are listed in Table 2.

The sensor directly provided the normalized difference vegetation index (NDVI) and ratio vegetation index (RVI), calculated as

NDVI = (R_NIR_ − R_Red_)/(R_NIR_ + R_Red_) (1)

RVI = R_NIR_/R_Red_ (2)

where R_NIR_ and R_Red_ represent near-infrared and red reflectance measured by the GreenSeeker sensor, respectively. Two time-normalized indices were further calculated using days after emergence (DAE). These indices were tested as simple operational indicators to evaluate whether dividing vegetation indices by crop age could reduce part of the growth-stage effect on spectral diagnosis. This normalization was not assumed to fully represent phenological development, but it allowed comparison of whether a simple time adjustment could improve the stability of NNI estimation:

TNDVI = NDVI/DAE(3)

TRVI = RVI/DAE(4)

### 2.3. Construction of the Critical Nitrogen Dilution Curve and Calculation of NNI

Critical N points used for descriptive visualization and conventional nonlinear fitting were identified separately for each experiment × sampling-date combination. For each combination, whole-plant dry matter was compared among N fertilizer rates using ANOVA followed by Tukey’s HSD test at *p* < 0.01. When dry matter responded significantly to N supply, the critical point was defined as the observation with the lowest plant N concentration among the N treatments whose dry matter was not significantly lower than the maximum dry matter observed at that sampling date. Sampling dates without a significant dry matter response to N, or without an identifiable non-N-limiting treatment, were excluded from the critical-point dataset. The selected critical N points were used only for descriptive visualization and for comparison with the conventional nonlinear fitting approach shown in the Results section; they were not the sole data used to estimate the Bayesian regional critical N dilution curve.

A Bayesian hierarchical model was used to estimate the regional potato critical N dilution curve, following Model 1 and the weakly informative prior structure proposed by Makowski et al. [8]. For sampling date i and N treatment j, measured biomass Wᵢⱼ was modeled with a linear–plateau response to plant N concentration Nᵢⱼ: Wᵢⱼ ~ Normal(µᵢⱼ, σ^2^W), where µᵢⱼ = BMAXᵢ + Sᵢ(Nᵢⱼ − NCᵢ) when Nᵢⱼ < NCᵢ, and µᵢⱼ = BMAXᵢ when Nᵢⱼ ≥ NCᵢ. This parameterization treats plant N concentration as the limiting variable controlling biomass accumulation before the plateau is reached, allowing the critical N concentration to be inferred from the transition between N-limited and non-N-limited growth. Compared with fitting a power function only to pre-selected critical points, this approach uses the full N response structure across N treatments and reduces the subjectivity associated with critical-point selection. The critical N concentration at sampling date i was linked to maximum biomass by NCᵢ = A1 × BMAXᵢ^−^ᴬ^2^. In Model 1, N_ij_ was modeled as Normal(NC_i_, σ^2^N), and weakly informative priors were assigned to A1, A2, BMAX_i_, S_i_, and residual variance components. The regional critical N dilution curve was obtained from the posterior distributions of A1 and A2 informed by the full dataset.

Posterior distributions were obtained using Markov chain Monte Carlo (MCMC) simulation implemented with the rjags package in R. Five MCMC chains were first run for 40,000 iterations each. After convergence was assessed, an additional 50,000 iterations were run to obtain posterior estimates. Convergence was evaluated using trace plots, the Gelman–Rubin potential scale reduction factor (Rhat), and effective sample size; convergence was considered acceptable when monitored parameters had Rhat < 1.10 and stable chain mixing. Posterior medians and 95% credible intervals were reported for A1, A2, and the fitted critical curve.

NNI was calculated as

NNI = PNC/Nc(5)

where PNC is the measured plant nitrogen concentration and Nc is the critical nitrogen concentration estimated from the regional critical N dilution curve. Because uncertainty in A1 and A2 directly affects Nc and hence NNI, NNI values close to the diagnostic threshold of 1.0 should be interpreted with caution. In operational applications, posterior draws of A1 and A2 can be used to recalculate Nc and NNI repeatedly and to express N-deficiency diagnosis as Pr(NNI < 1), rather than relying only on a single point estimate.

The purpose of this study was to establish a regional critical N dilution curve for major potato-producing areas in northern China rather than cultivar-specific curves. Therefore, valid observations from different years, sites, and the two representative cultivars were pooled to estimate the overall curve parameters. This approach expanded the observed range of whole-plant dry matter and plant N concentration and improved the regional applicability of the curve. Because cultivar effects were significant in the agronomic dataset, the resulting curve should be interpreted as a regional average diagnostic reference within the scope of the cultivars and environments tested.

### 2.4. Statistical Analysis

The relationships between vegetation indices and measured NNI were evaluated using treatment-mean observations in both stage-specific and pooled analyses. At each sampling stage, bivariate screening analyses were conducted to evaluate the association between NNI and each vegetation index (NDVI, RVI, TNDVI, and TRVI).

Because sampling dates differed slightly among experiments, nearby dates representing similar developmental phases were grouped for stage-level reporting: 35, 37, and 40 DAE were grouped as 35–40 DAE; 70 and 71 DAE were grouped as 70–71 DAE; and 77 and 78 DAE were grouped as 77–78 DAE. The number of treatment-mean observations and coefficient of determination (R^2^) were used to compare the relative performance of the four vegetation indices across sampling stages. These stage-specific analyses were used to identify suitable diagnostic stages and indices, rather than as final deployment equations for field recommendation. All sampling stages were then pooled to develop whole-season regression models between vegetation indices and NNI. The pooled whole-season regressions were included only as baseline models to test whether growth-stage-agnostic empirical relationships could adequately estimate NNI; they were not intended as recommended diagnostic equations for field application. Bayesian hierarchical modeling was conducted using R (version 4.3.2; R Core Team, Vienna, Austria) [22] and RStudio (version 2023.12.0; Posit Software, Boston, MA, USA) [23], and JAGS was called through the jags.model function in the rjags package (version 4-17) [24]. Analysis of variance was performed using SPSS 25.0 after testing for normality and homogeneity of variance. Treatment differences were evaluated using Tukey’s HSD test at *p* < 0.01. For ANOVA outputs, degrees of freedom, F values, and exact *p* values were retained for detailed reporting; in compact ANOVA summary tables, * and ** indicate significance at *p* < 0.05 and *p* < 0.01, respectively. Nonlinear regression and figure preparation were conducted using Origin 2021.

## 3. Results

### 3.1. Responses of Whole-Plant Dry Matter and Plant Nitrogen Concentration to Nitrogen Fertilization

Analysis of variance showed that year, site, N rate, and cultivar had highly significant effects on whole-plant dry matter and plant N concentration at all growth stages (*p* < 0.01) (Table 3). This indicates that the field experiments generated a wide gradient of crop N status, ranging from N deficiency to excessive N supply, thereby providing a suitable dataset for model development.

**Figure 1:**
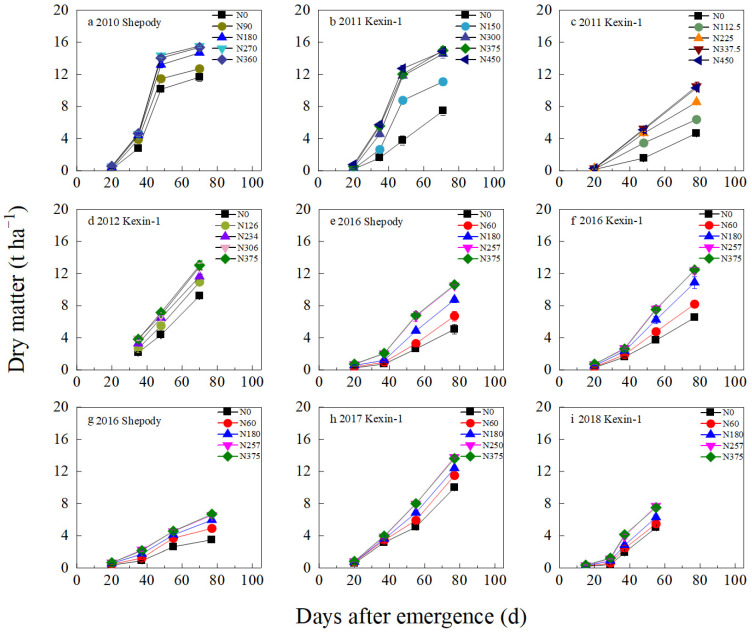
Effects of nitrogen application rate on the dynamics of whole-plant dry matter in potato across nine field experiments in northern China. Each panel represents one experiment: (**a**) Exp.1, 2010, Shepody; (**b**) Exp.2, 2011, Kexin 1; (**c**) Exp.3, 2011, Kexin 1; (**d**) Exp.4, 2012, Kexin 1; (**e**) Exp.5, 2016, Shepody; (**f**) Exp.6, 2016, Kexin 1; (**g**) Exp.7, 2016, Shepody; (**h**) Exp.8, 2017, Kexin 1; and (**i**) Exp.9, 2018, Kexin 1. Points are treatment means, error bars indicate standard errors, and line-end labels indicate N fertilizer rates in kg N ha^−1^.

As shown in Figure 1, whole-plant dry matter increased with increasing N rate across cultivars and experimental sites. Differences among N treatments were relatively small at early growth stages but became more pronounced as growth progressed, particularly during the tuber bulking stage. Whole-plant dry matter was consistently lowest under the zero-N treatment. At higher N rates, dry matter accumulation tended to level off, indicating that N was no longer the primary limiting factor for growth.

**Figure 2:**
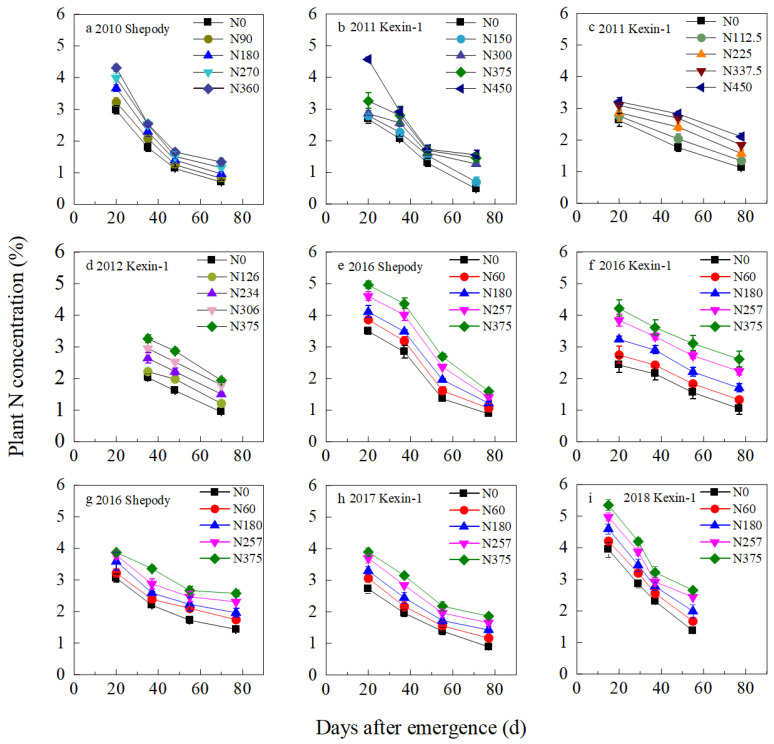
Effects of nitrogen application rate on the dynamics of whole-plant nitrogen concentration in potato across nine field experiments in northern China. Panel definitions are the same as in Figure 1. Points are treatment means, error bars indicate standard errors, and line-end labels indicate N fertilizer rates in kg N ha^−1^.

Plant N concentration also increased with N rate (Figure 2). Across the growing season, plant N concentration generally declined with increasing plant dry matter, consistent with the N dilution effect during crop growth. High-N treatments maintained higher plant N concentrations throughout the growing season, whereas low-N treatments showed a more rapid decline. Similar trends were observed for the two cultivars.

### 3.2. Establishment and Evaluation of the Critical Nitrogen Dilution Curve

**Figure 3:**
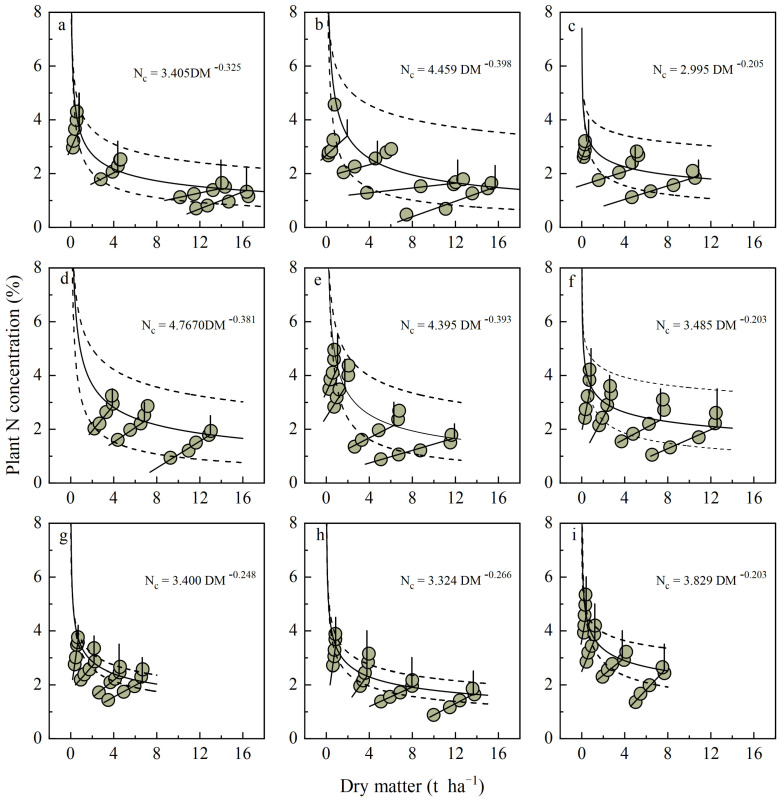
Experiment-specific critical nitrogen dilution curve for potato across the nine field experiments. Each panel represents one experiment: (**a**) Exp.1, 2010, Shepody; (**b**) Exp.2, 2011, Kexin 1; (**c**) Exp.3, 2011, Kexin 1; (**d**) Exp.4, 2012, Kexin 1; (**e**) Exp.5, 2016, Shepody; (**f**) Exp.6, 2016, Kexin 1; (**g**) Exp.7, 2016, Shepody; (**h**) Exp.8, 2017, Kexin 1; and (**i**) Exp.9, 2018, Kexin 1. Open circles represent treatment means at each N fertilizer rate and sampling date, and vertical bars indicate standard errors of plant N concentration. Solid lines show the fitted experiment-specific critical nitrogen dilution curve, and dashed lines indicate the uncertainty envelope.

**Figure 4:**
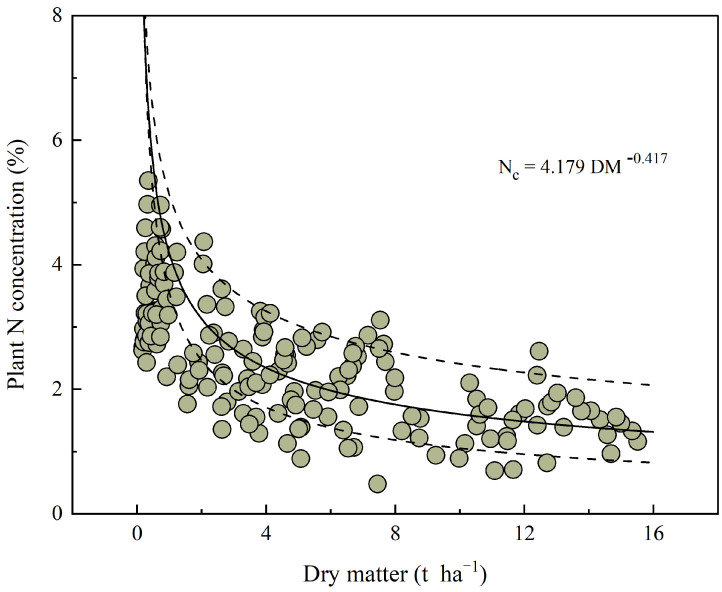
Bayesian regional critical nitrogen dilution curve for potato based on pooled treatment means from all nine field experiments. Open circles represent pooled treatment means across N fertilizer rates and sampling dates. The solid line represents the regional critical nitrogen dilution curve, Nc = 4.179 × DW^−0.417^, where DW is whole-plant dry matter. Dashed lines indicate the uncertainty envelope around the regional curve.

**Figure 5:**
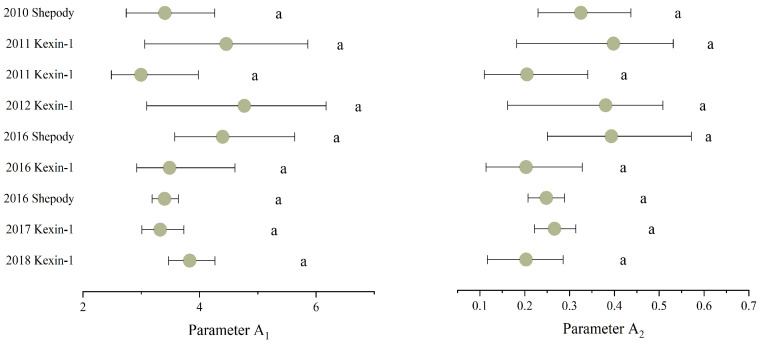
Posterior median estimates and 95% credible intervals of the experiment-specific parameters A1 and A2 in the Bayesian critical nitrogen dilution model Nc = A1 × DW^−^ᴬ^2^. Points represent posterior medians, horizontal error bars represent 95% credible intervals, and *y*-axis labels identify the nine experiments. Different lowercase letters indicate significant differences among experiments at *p* < 0.01 according to Tukey’s HSD test; identical letters indicate no significant differences.

The critical N dilution curve developed from the nine field experiments is shown in Figure 3 and Figure 4. Across years, sites, and cultivars, the minimum N concentration required to sustain maximum growth declined with increasing whole-plant dry matter (DW), following a power function relationship (Figure 3). The posterior medians and 95% credible intervals of parameters A1 and A2 obtained from the Bayesian model are shown in Figure 5.

The fitted critical N dilution curve was: Nc = 4.179 × DW^−0.417^, where Nc is the critical N concentration, and DW is whole-plant dry matter. The uncertainty envelope around the regional curve was relatively narrow (Figure 4), and the posterior medians and 95% credible intervals of A1 and A2 are shown in Figure 5. These results indicate that the curve can be used as a regional average diagnostic reference within the tested environments, rather than as a cultivar-specific diagnostic standard. Therefore, the curve was used as the basis for calculating potato NNI and evaluating plant N status under different N fertilizer treatments.

### 3.3. Relationships Between Vegetation Indices and NNI at Different Growth Stages

The ability of spectral indices to estimate potato NNI varied markedly with growth stage (Table 4). At 15–20 DAE, correlations between vegetation indices and NNI were generally weak, suggesting that canopy spectral indices were not reliable for diagnosing potato N status at the early seedling stage. This was likely due to limited canopy coverage and strong soil background effects.

From 29 to 70 DAE, the relationships between vegetation indices and NNI became stronger, indicating that this period was the main effective window for spectral diagnosis of potato N status. At 29 DAE, all four indices showed strong relationships with NNI, with R^2^ values ranging from 0.878 to 0.888. At 35–48 DAE, most indices maintained moderate to strong relationships with NNI. At 55 DAE, RVI showed the strongest relationship with NNI, with an R^2^ of 0.853. At 70–71 DAE, NDVI and RVI still showed moderate relationships with NNI, with R^2^ values of 0.498 and 0.644, respectively. In contrast, TNDVI and TRVI showed weaker stability at later growth stages.

For field deployment, GreenSeeker-based NNI estimation should be used as a stage-specific diagnostic tool rather than as a whole-season equation. Quantitative diagnosis is not recommended at 15–20 DAE because limited canopy cover and soil background effects weaken the spectral signal. The primary operational window is 29–55 DAE, when RVI showed the strongest and most consistent relationship with NNI. Measurements around 70 DAE can provide auxiliary diagnostic information for NDVI and RVI, but should be interpreted cautiously due to canopy senescence and N remobilization. Quantitative spectral diagnosis at 77–78 DAE or later is not recommended. Thus, the broader effective phenological window may be described as approximately 29–70 DAE.

At 78 DAE, all indices had R^2^ values below 0.10, indicating that canopy senescence and N remobilization weakened the ability of spectral indices to reflect whole-plant N status. Overall, the tuber initiation–bulking period, approximately 29–70 DAE under the tested environments, can be regarded as the appropriate diagnostic window for spectral estimation of potato NNI, with RVI showing relatively consistent performance across multiple stages (Table 4).

**Figure 6:**
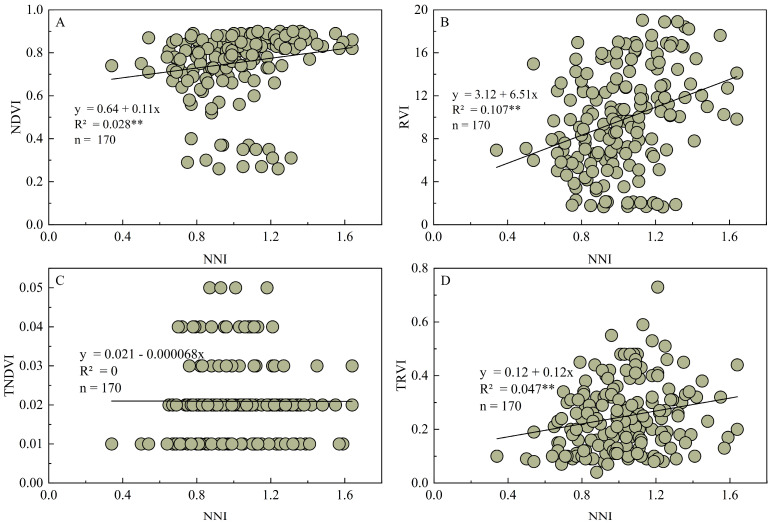
Relationships between vegetation indices and nitrogen nutrition index (NNI) using 170 observations pooled across sampling stages from the 2010–2018 experiments. Panels show (**A**) NDVI, (**B**) RVI, (**C**) TNDVI, and (**D**) TRVI. NNI is shown on the *x*-axis, and the corresponding vegetation index is shown on the *y*-axis. Solid lines indicate simple linear regressions, and each panel reports the fitted equation, coefficient of determination (R^2^), and sample size (*n*). Double asterisks indicate that the fitted linear regression is significant at *p* < 0.01.

Figure 6 shows the relationships between vegetation indices and NNI when all sampling stages were pooled. Overall, the relationships were weak. RVI had the highest coefficient of determination among the tested indices, but its R^2^ was only 0.107, although the relationship was highly significant. This indicates that RVI was the most responsive index in the pooled dataset but explained only 10.7% of the variation in NNI. NDVI and TRVI had R^2^ values of 0.028 and 0.047, respectively, whereas TNDVI showed almost no linear relationship with NNI. These pooled regressions therefore serve as baseline models demonstrating that a single whole-season linear equation is insufficient for quantitative diagnosis of potato NNI. Practical spectral diagnosis of potato N status should account for growth stage and should focus on the effective phenological window corresponding to tuber initiation and tuber bulking, approximately 29–70 DAE under the tested environments.

## 4. Discussion

### 4.1. Rationality and Applicability of the Regional Critical Nitrogen Dilution Curve

Based on nine field experiments conducted across major potato-producing areas in northern China, this study developed the critical N dilution curve Nc = 4.179 × DW^−0.417^. The curve indicates that the critical N concentration required to sustain maximum growth decreased with increasing whole-plant dry matter, which is consistent with the theoretical basis of critical N dilution [5,6]. Physiologically, potato growth involves a gradual shift in dry matter accumulation from vegetative organs to tubers, together with changes in the proportion of structural and storage tissues. As a result, whole-plant N concentration declines as biomass increases. Therefore, the curve obtained in this study is not only statistically meaningful but also consistent with the N dilution and organ allocation processes of potatoes. Giletto et al. also showed that potato N dilution is closely related to shoot growth, tuber formation, and N allocation among organs [2].

Compared with previously reported potato critical N dilution curves, the parameters obtained in this study were within the range reported in the literature. The main value of this curve is not that it provides a fundamentally different equation, but that it was developed using multi-year, multi-site, multi-N-rate data from two representative cultivars in northern China. This makes it more relevant as a regional diagnostic reference for field N management. Previous studies have shown that potato critical N curves may be affected by environmental conditions, growing season, sampled organ, cultivar, and management practices [2,3,11,20]. Therefore, external curve parameters should not be applied directly without local calibration and validation. The regional curve developed here addresses this need by providing a diagnostic reference based on the target production region.

The Bayesian hierarchical model was useful mainly because it enabled parameter estimation while explicitly representing uncertainty. Conventional approaches usually identify critical N points first and then fit a nonlinear regression curve, which can be affected by subjective point selection, sample distribution, and the number of experiments. Because the present dataset included different years, sites, and cultivar combinations, the estimated curve parameters were inevitably influenced by variation among experimental sources. Compared with ordinary nonlinear regression, a Bayesian hierarchical model can estimate the overall curve while quantifying parameter uncertainty, making it more suitable for multi-environment datasets [8,10]. Studies on potato have also shown that hierarchical Bayesian methods can describe the uncertainty of critical N curve parameters under genotype × environment × management interactions [11]. Thus, the purpose of using a Bayesian approach in this study was not to eliminate differences among years, sites, or cultivars, but to establish a more robust regional average diagnostic reference.

It should also be emphasized that the Bayesian approach does not automatically remove cultivar differences. Table 3 showed that cultivar had significant effects on whole-plant dry matter and plant N concentration. However, this does not necessarily mean that completely separate critical N dilution curves are required for each cultivar. The objective of this study was to develop an overall diagnostic curve for regional application, rather than cultivar-specific curves. Pooling data from two commonly grown cultivars in the study region expanded the observed range of DW and PNC and improved the operational applicability of the curve at the regional scale. Nevertheless, the curve should be interpreted as a regional average diagnostic reference within the scope of this dataset. For cultivars with markedly different genetic backgrounds, or for studies aiming to compare cultivar-specific N use efficiency, cultivar-specific parameter estimation and independent validation will still be necessary.

### 4.2. Performance of RVI for Estimating NNI and Limitations of Whole-Season Modeling

Among the four vegetation indices evaluated in this study, RVI showed relatively strong relationships with NNI at multiple sampling dates, especially at 29, 35, 48, 55, and 70 DAE. RVI is calculated as the ratio of near-infrared to red reflectance. Red reflectance is closely associated with chlorophyll absorption, whereas near-infrared reflectance is influenced by canopy structure, leaf area, and cellular structure. Because NNI integrates both plant N concentration and biomass information, indices that respond to both chlorophyll absorption and canopy structure are more likely to be related to NNI. Previous studies have also shown that red and near-infrared reflectance combinations can reflect canopy chlorophyll and structural characteristics, and that vegetation indices differ in their sensitivity to leaf area and canopy chlorophyll density [13,14].

The relatively better performance of RVI in this study may be related to the saturation tendency of NDVI under high canopy coverage. During tuber initiation and tuber bulking, the potato canopy expands rapidly, and leaf area and biomass increase substantially. Under these conditions, NDVI may become less sensitive, whereas RVI may retain a wider response range. Therefore, RVI showed more stable responses to NNI within the key diagnostic window of 29–70 DAE. However, this does not imply that RVI is superior to NDVI at all growth stages. For example, Table 4 shows that NDVI also had relatively high correlations with NNI at certain sampling dates. A more appropriate interpretation is that RVI performed best overall within the diagnostic window identified in this study, but it should not be considered a universal index for all stages and conditions.

The pooled whole-season models further demonstrate the limitation of ignoring the growth stage. When all sampling stages were combined, RVI showed the highest R^2^ among the tested indices, but the value was only 0.107. NDVI and TRVI had R^2^ values of 0.028 and 0.047, respectively, and TNDVI showed almost no linear relationship with NNI. These results indicate that growth-stage variation substantially weakened the overall linear relationship between vegetation indices and NNI. At early stages, low canopy coverage and soil background effects influenced the spectral signal. During tuber initiation and bulking, canopy structure became more stable, and N differences were more clearly reflected in spectral responses. At later stages, leaf senescence and N remobilization to tubers altered the relationship between canopy reflectance and whole-plant N status. Therefore, the low R^2^ of the pooled models is not merely a statistical limitation; it indicates that a growth-stage-agnostic equation should not be deployed for real-season N monitoring. RVI should be interpreted as a stage-specific indicator within the appropriate phenological window rather than as a whole-season predictor. Recent studies on potato N remote sensing have also indicated that NNI can serve as a useful link between physiological diagnosis and remote sensing, but model stability depends on growth stage, sensing platform, and ground validation [16,17,19]. The present results therefore support the use of RVI for rapid field diagnosis during 29–70 DAE, but do not support pooling all growth stages into a single empirical model.

### 4.3. Physiological Interpretation of the Spectral Diagnostic Window

The spectral diagnosis of potato NNI showed strong growth-stage dependence. At 15–20 DAE, the relationships between vegetation indices and NNI were weak, indicating that early seedling stages are not suitable for stable spectral diagnosis. At this stage, the plants were small, canopy coverage was limited, and the sensor signal was strongly affected by soil background and uneven plant distribution. Even when plant N concentration differed among N treatments, canopy-scale reflectance may not have captured those differences reliably. Yu et al. also reported that GreenSeeker-based spectral diagnosis of potato N status was affected by soil background at early growth stages, whereas the relationship between sensor indices and plant N status became stronger as the canopy developed [25]. Similarly, Tang et al. used hyperspectral data for potato nitrogen nutrition diagnosis and further demonstrated the potential of spectral information for assessing potato N status [26].

The main diagnostic window identified in this study was 29–70 DAE. This period generally corresponded to tuber initiation and tuber bulking, when canopy closure progressed, leaf area and dry matter increased rapidly, and differences in N supply were more clearly expressed through chlorophyll content, canopy structure, and biomass accumulation. As a result, NDVI, RVI, and related indices showed stronger relationships with NNI. At 29 DAE, all four indices had R^2^ values close to or above 0.88, suggesting clear spectral separation among N treatments. From 35 to 55 DAE, RVI maintained relatively high correlations with NNI, indicating good diagnostic stability during the early and middle phases of tuber development.

At 70–71 DAE, the performance of different indices began to diverge. NDVI and RVI still showed moderate relationships with NNI, indicating that they retained some diagnostic value at this stage. In contrast, TNDVI and TRVI decreased markedly, suggesting that simple normalization by DAE did not improve index stability at later stages. By 78 DAE, all indices had R^2^ values below 0.10. This indicates that leaf senescence, canopy structural degradation, and N remobilization to tubers substantially weakened the ability of canopy reflectance to represent whole-plant N status. Therefore, the 29–70 DAE diagnostic window identified here should be interpreted as an approximate phenological window, with 29–55 DAE as the primary operational window and 70 DAE as an auxiliary diagnostic stage. Extending the diagnosis to 78 DAE is not recommended.

The results for TNDVI and TRVI also show that simple normalization by DAE is insufficient to characterize potato growth progress. DAE was used in this study because it is operationally simple and can be readily recorded in field management. However, DAE should not be interpreted as a fully interchangeable developmental scale across years, sites, and cultivars. Developmental rates may differ among environments, years, sites, and cultivars, and the same DAE may not correspond to the same canopy closure, tuber initiation, or tuber bulking status. Therefore, the 29–70 DAE diagnostic window identified here should be interpreted as an approximate phenological window corresponding mainly to tuber initiation and tuber bulking, rather than as a rigid calendar-day rule. Future studies may improve model stability by incorporating accumulated temperature, phenological stage, canopy coverage, or leaf area index rather than relying solely on DAE. Sun et al. also emphasized that canopy structure and growth stage are key factors affecting the stability of remote sensing models for potato traits [15].

### 4.4. Application, Limitations, and Future Research

The practical contribution of this study is that it provides a relatively simple pathway for field diagnosis of potato N status. First, NNI is calculated using a regional critical N dilution curve. Second, spectral indices are evaluated for their ability to directly estimate NNI. Finally, the diagnostic window is defined to avoid applying the spectral model outside its effective growth stages. Compared with conventional methods based on destructive sampling and laboratory analysis, this approach is more suitable for rapid field monitoring and in-season fertilization decisions. Compared with UAV or satellite-based approaches, GreenSeeker-based proximal sensing is operationally simple, less affected by illumination conditions, and suitable for repeated measurements at the field scale. Recent studies indicate that potato N remote sensing is moving from single-index estimation toward UAV-based spatial diagnosis of NNI and machine-learning-assisted multi-source data fusion for in-season N status prediction [16,19,27]. Nevertheless, for practical production, proximal sensing remains attractive because of its low cost, operational simplicity, and straightforward interpretation.

Several limitations should be acknowledged. First, although the experiments covered multiple years, sites, and N rates, they were conducted within major potato-producing areas in northern China and included only two representative cultivars. Therefore, the critical N dilution curve developed here should be considered a regional average diagnostic reference. Its applicability to other climate zones, soil types, cropping systems, and cultivars requires further validation. Second, the spectral indices used in this study were based on red and near-infrared bands from GreenSeeker. The use of only red and near-infrared bands limits the ability of the sensor to separate chlorophyll absorption, canopy structural variation, soil background, and late-season N remobilization. This limitation is particularly important after canopy senescence begins, when canopy reflectance no longer represents whole-plant N status reliably. Red-edge bands, hyperspectral features, thermal information, canopy coverage, leaf area index, and three-dimensional canopy structural information may improve diagnosis beyond the main tuber bulking period.

In addition, the present stage-specific analyses were designed as transparent screening analyses, but they do not formally test whether the stage effect changes continuously or at discrete breakpoints. Segmented, nonlinear, or stage-aware models that include vegetation index, phenological stage, accumulated temperature, and their interactions should be evaluated in future work. Such models may better represent the biological transition from early canopy establishment to tuber bulking and senescence. For operational NNI diagnosis, uncertainty propagation should also be extended from the Bayesian critical N curve to downstream NNI classification, especially for observations close to NNI = 1. Operationally, NNI estimates close to the threshold of 1.0 should be treated as borderline cases. For these, Pr(NNI < 1) can provide a more informative decision metric and should be considered together with growth stage and repeated canopy measurements.

In addition, this study focused on NNI as the diagnostic target and did not quantify the relationships between NNI and tuber yield, dry matter content, starch concentration, or processing quality. Sandaña et al. suggested that NNI can be used to evaluate differences in N use efficiency among potato genotypes [28]. However, the present study was not designed for cultivar screening and did not develop cultivar-specific critical N dilution curves. Therefore, the regional curve developed here is more suitable for field N diagnosis than for cultivar selection or genotype-specific N use efficiency evaluation. Future research should expand the range of cultivars and environments, integrate Bayesian updating and multi-source sensing, and link NNI diagnosis with yield, quality, and fertilizer recommendation to develop a complete pathway from N diagnosis to N management decisions.

## 5. Conclusions

This study established a regional critical nitrogen dilution curve for potato in northern China using multi-year, multi-site field data and a Bayesian hierarchical model. The curve provides a practical reference for calculating NNI and evaluating crop nitrogen status under local production conditions. Among the spectral indices examined, RVI showed the most consistent relationship with NNI during tuber initiation and bulking. The primary operational window for quantitative diagnosis was 29–55 DAE, whereas measurements around 70 DAE may provide auxiliary information. The weak performance of pooled whole-season models demonstrates that a single linear relationship across all growth stages is inadequate for reliable NNI estimation. Spectral diagnosis should therefore be restricted to appropriate phenological windows and interpreted in relation to canopy development, with near-threshold NNI values treated as uncertain or borderline cases. Further work should validate the proposed curve and spectral approach across broader cultivar, environmental, and management conditions, and should integrate nitrogen diagnosis with yield, quality, and fertilizer recommendation frameworks.

## Figures and Tables

**Table 1:** Soil physicochemical properties before planting.

Experiment	Year	Cultivar	Site	O.M. g/kg	Total N g/kg	Avail P mg/kg	Avail K mg/kg	pH
Exp.1	2010	Shepody	Wuchuan County	16.1	0.89	12.2	141	8.4
Exp.2	2011	Kexin 1	Wuchuan County	13.4	1.12	13.1	162	8.1
Exp.3	2011	Kexin 1	Linxi County	22.3	1.35	21.3	185	7.7
Exp.4	2012	Kexin 1	Wuchuan County	14.5	1.08	10.7	103	8.2
Exp.5	2016	Shepody	Chahar Right Middle Banner	18.8	1.40	12.5	196	8.0
Exp.6	2016	Kexin 1	Chahar Right Middle Banner	18.8	1.40	12.5	196	8.0
Exp.7	2016	Shepody	Hanggin Rear Banner	7.4	0.40	5.1	179	8.9
Exp.8	2017	Kexin 1	Chahar Right Middle Banner	16.8	1.50	12.6	117	8.1
Exp.9	2018	Kexin 1	Chahar Right Middle Banner	21.3	1.46	10.5	124	8.0

O.M., organic matter; Total N, total nitrogen; Available P, available phosphorus; Available K, available potassium. Organic matter, total N, available P, and available K are expressed in g kg^−1^, g kg^−1^, mg kg^−1^, and mg kg^−1^, respectively, unless otherwise stated.

**Table 2:** Experimental design, N fertilizer rates, and sampling dates.

Experiment	N Treatment	Sampling Dates (DAE)	Code
Exp.1	0, 90, 180, 270, 360	20, 35, 48, 70	a
Exp.2	0, 150, 300, 375, 450	20, 35, 48, 71	b
Exp.3	0, 113, 225, 338, 450	20, 48, 78	c
Exp.4	0, 126, 234, 306, 375	35, 48, 70	d
Exp.5	0, 60, 180, 257, 375	20, 37, 55, 77	e
Exp.6	0, 60, 180, 257, 375	20, 37, 55, 77	f
Exp.7	0, 60, 180, 257, 375	20, 37, 55, 77	g
Exp.8	0, 60, 180, 257, 375	20, 37, 55, 77	h
Exp.9	0, 60, 180, 257, 375	15, 29, 40, 55	i

DAE indicates days after emergence. Nitrogen treatments are expressed in kg N ha^−1^.

**Table 3:** Effects of year, site, N rate, and cultivar on potato whole-plant dry matter and plant N concentration.

Sources of Variance	Dry Matter (t ha^−1^)				Plant N Concentration (%)			
	Seedling Stage	Tuber Initiation Stage	Tuber Bulking Stage	Starch Accumulation Stage	Seedling Stage	Tuber Initiation Stage	Tuber Bulking Stage	Starch Accumulation Stage
Year	**	**	**	**	**	**	**	**
Site	**	**	**	**	**	**	**	**
N rate	**	**	**	**	**	**	**	**
Cultivar	**	**	**	**	**	**	**	**

Note: ** indicates a significant effect at *p* < 0.01.

**Table 4:** Sample sizes and coefficients of determination between vegetation indices and NNI at different sampling dates.

DAE	*n*	R^2^			
		NDVI	RVI	TNDVI	TRVI
15	5	0.278	0.319	0.034	0.253
20	35	0.034	0.123	0.034	0.145
29	5	0.878	0.888	0.886	0.881
35–40	40	0.555	0.579	0.546	0.532
48	20	0.510	0.679	0.670	0.579
55	25	0.671	0.853	0.177	0.393
70–71	15	0.498	0.644	0.098	0.144
77–78	25	0.093	0.068	0.094	0.068

Note: *n* indicates the number of treatment-mean observations used in each stage-specific screening analysis. Because sampling dates differed slightly among experiments, nearby dates representing similar developmental phases were grouped for reporting: 35, 37, and 40 DAE were grouped as 35–40 DAE; 70 and 71 DAE were grouped as 70–71 DAE; and 77 and 78 DAE were grouped as 77–78 DAE. R^2^ values were used as descriptive indicators of the strength of stage-specific bivariate relationships between NNI and each vegetation index. These analyses were used to compare the relative performance of vegetation indices and identify the diagnostic window, rather than as final field deployment equations.

## Data Availability

The data presented in this study are available on request from the corresponding author. The data are not publicly available because they originate from multi-year field experiments and institutional project datasets.

## Abbreviations

The following abbreviations are used in this manuscript:

N	Nitrogen
DW	Whole-plant dry matter
PNC	Plant nitrogen concentration
NDVI	Normalized difference vegetation index
RVI	Ratio vegetation index
TNDVI	Time-normalized difference vegetation index
TRVI	Time-normalized ratio vegetation index
DAE	Days after emergence
MCMC	Markov chain Monte Carlo
CI	Credible interval
O.M.	Organic matter
LAI	Leaf area index
JAGS	Just Another Gibbs Sampler
NNI	Nitrogen nutrition index
